# Systematic Review of Survival Outcomes of Pregnancy-Associated Breast Cancer in Asian Countries

**DOI:** 10.21315/mjms2024.31.2.2

**Published:** 2024-04-23

**Authors:** Syed Sana Abrar, Norsa’adah Bachok

**Affiliations:** Unit of Biostatistics and Research Methodology, School of Medical Sciences, Universiti Sains Malaysia, Kelantan, Malaysia

**Keywords:** pregnancy-associated breast cancer, breast cancer and pregnancy, survival outcomes, systematic review

## Abstract

Pregnancy-associated breast cancer (PABC) is a rare type of gestational cancer. It poses a significant challenge in diagnosis and management, especially in Asian countries with limited resources. We carried out a systematic literature review and narrative synthesis to identify survival outcomes for women with PABC in Asia. We searched MEDLINE, PubMed, Cochrane Library and the reference lists of the included English language articles for those conducted between January 2010 and August 2022. The search terms were pregnancy-associated breast cancer, breast cancer AND pregnancy, survival of PABC and prognosis of PABC patients. PABC is defined as breast cancer diagnosed either during pregnancy or 1 year–5 years postpartum. This review included observational studies conducted in Asian countries. The final 11 articles met the selection criteria and were analysed. Five of the studies had high quality methods as assessed using the Joanna Briggs Institute (JBI) checklist. We reported study design, year of diagnosis, country, definition of PABC, control group, age of participants, median follow-up time, survival outcomes and pregnancy as prognostic factors. Only five studies reported that PABC patients had a poor overall or disease-free survival rate compared to the control. Pregnancy was a significant independent prognostic factor of breast cancer in only two studies. This review highlights that pregnancy has an unconfirmed association with breast cancer survival in Asia. Most studies that found a non-significant association had small samples, thus there is a need for large-scale multinational epidemiological studies in Asia to establish the survival outcomes in PABC patients.

## Introduction

Although the incidence of breast cancer in Asia is lower than in North America, Western Europe and Oceania, the rates have increased over the past decades. Breast cancer in Asian countries now accounts for 40% of all diagnosed cases globally, and its mortality has similarly increased among Asian women (1). Over 600,000 new cases of breast cancer were reported in Asia in 2012, making breast cancer the most frequent cancer in Asian women, accounting for 21.2% of all cancer cases in women (2). With an average age standardised incidence rate (ASIR) of 29.1 per 100,000 population, breast cancer incidence rates in Asian countries are expected to be a quarter to a third of those in countries historically high risk (3). India, followed by Pakistan, had the highest incidence rates of breast cancer in Southern Asia (4), while Japan and South Korea topped in Eastern Asia (5). Lebanon had the highest ASIR of 78.7 in West Asia (6) and Singapore had the most recorded cases in Southeast Asia (7).

Compared to European countries, mortality associated with breast cancer was much higher in Asian countries, with a mortality rate of 231.0 compared to 131.3 (8). Breast cancer deaths accounted for an estimated 231,013 women in Asia in 2012, accounting for 7% of all deaths and 40.8% of cancer deaths, ranking second after lung cancer in women (2). On the other hand, the mortality-to-incidence ratios in Asia were substantially greater than in Western countries. Most Asian countries are low- and middle-income countries, where breast cancer occurred at a younger age and at a later stage, and patients were more likely to die from the disease than in Western countries (2).

The age at diagnosis in Asian countries was lower than in high-income countries, with a study detecting 15.5% of breast cancer cases under the age of 40 years old in Korean women (9), another reporting the mean age to be 41.8 years old in Bangladesh (10), and the median age of breast cancer diagnosis to be 44 years old in Jordan (11), 45 years old in Saudi Arabia, (12) and 48 years old–50 years old in China (5).

Breast cancer that develops during or within a year of pregnancy is known as pregnancy-associated breast cancer (PABC) (13). PABC is also defined as breast cancer diagnosed within 24 months after childbirth (14) or within 5 years postpartum (15). The definition of PABC varies according to the length of the postpartum period, which may lead to different conclusions about the relationship between pregnancy, postpartum period and breast cancer. PABC is also known as gestational breast cancer. PABC occurs in women of reproductively active age and at relatively younger age than in the general breast cancer population.

PABC is a rare condition with limited research from low- and middle-income countries. PABC is uncommon, with an incidence rate between 17.5 and 39.9 per 100,000 births (16). The occurrence is much lower during pregnancy than during the post-partum period. A single institution in New York reported that the incidence of PABC was 2.1% over the period 2009–2018 (14). The frequency of PABC is expected to increase as maternal age continues to rise, from 26.0 years in 1982 to 27.4 years in 2002 (17).

There was an unfavourable link between pregnancy and breast cancer, noting that less than 20% of pregnant women with breast cancer lived more than 5 years after diagnosis (18). Several studies from Western countries have analysed the survival outcomes in women with PABC; however, similar studies were scarce in Asia. Therefore, the aim of this systematic review was to review the survival outcomes of PABC patients in Asia. We hypothesised that pregnancy is a poor prognostic factor for survival among women with breast cancer.

## Method

### Search Strategy

This review was conducted in accordance with the Preferred Reporting Items for Systematic Reviews and Meta-Analyses (PRISMA) guidelines (19). We searched MEDLINE, PubMed, Cochrane Library and the reference lists of the included studies. We used the Boolean operator ‘AND’ and limited the search to a date range between 2010 and 2022. The search terms were pregnancy-associated breast cancer, breast cancer AND pregnancy, survival of PABC and prognosis of PABC.

### Eligibility Criteria

The search was restricted to original articles conducted in Asian countries published in English language. Only observational study designs and available full articles were included in the review. The exclusion criteria were studies with fewer than 10 patients, article abstracts, literature review, books, letters to editor and studies published in proceedings. PABC was defined as breast cancer diagnosed during pregnancy or within 5 years postpartum.

### Study Selection Process

A title reading was the first step used to detect pertinent articles. The abstract of those articles considered relevant was retrieved and further scrutinised. Duplicate articles and those for which the full text was unobtainable or those which did not fulfil the selection criteria were excluded. Articles that lacked information on the survival and prognosis of PABC were also excluded. Finally, all the included full text articles were analysed narratively.

### Quality Assessment

The methodological quality of selected articles that met the review criteria was assessed using the checklist provided by the Joanna Briggs Institute (JBI) (20). It includes 11 questions on population, exposure, measurements, confounders, outcomes, follow-up and statistical analysis. We assigned scores of the checklist to determine the overall methodological quality of the articles. A score of 0 indicates ‘not reported’ or ‘unclear’ and a score of 1 indicates ‘satisfactory’ for the checklist item. Articles with a score ≥ 75% were considered high quality and those with a score ≤ 25% as low quality, with the reminder considered moderate quality.

### Data Collection and Analysis

The researchers independently screened the titles and abstracts and finally evaluated the full text of the selected articles and entered the information into a spreadsheet. Disagreements were resolved through discussion. Information from the included studies was extracted by one author and verified by another. Data included were study design, year of diagnosis, country, sample size, pregnancy time, PABC definition, control group, age at diagnosis, follow-up time and survival outcomes. We reported the findings as the percentage of overall survival (OS), disease-free survival (DFS) and event-free survival (EFS), median survival time, adjusted hazard ratio (aHR), 95% confidence interval (CI) and *P*-value.

## Results

### Description and Characteristics of Included Articles

**Figure 1:**
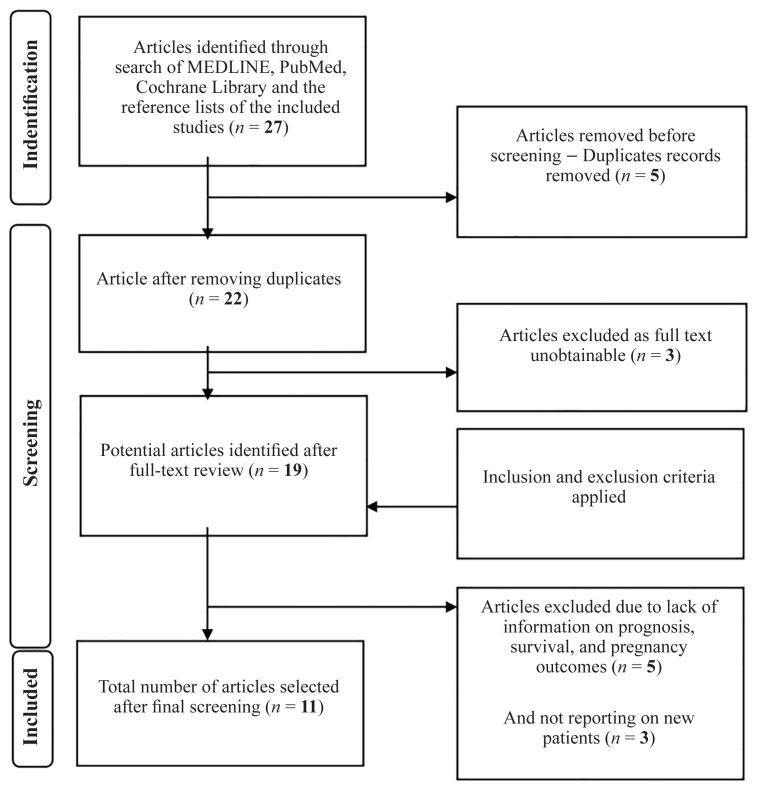
Flow diagram of the study process

A flowchart of the review process is shown in Figure 1. A total of 27 articles were identified from searching the databases. Five duplicate articles and three articles for which the full text was unobtainable were removed. Then, five articles were excluded due to a lack of information on survival and prognosis, and three were excluded due to reporting the same patients. Finally, 11 articles were included in the qualitative analysis. The JBI checklist for methodological quality assessment showed that five articles were of high quality and six articles were of moderate quality.

Table 1 summarises the characteristics and the survival and prognostic outcomes of the selected articles. Most (72.7%) of the articles were published between 2018 and 2021 (21–28) and three of them were published in 2015 or earlier (29–31). All studies were retrospective cohort studies that reviewed medical records. Only two studies used data from a national cancer registry (27, 28), one study had data from 27 hospitals (22) and others were single hospital data. The largest study consisted of 2,430 PABC patients from the Taiwan Cancer Registry from 2002–2014 (28). The smallest series presented 26 PABC patients from a medical centre in Taiwan (30). The 11 included studies were from six countries: China (4), South Korea (2), Taiwan (2), Japan (1), India (1) and Saudi Arabia (1). Four studies did not mention whether the PABC patients were diagnosed antepartum or postpartum (24, 26, 27, 29).

### Clinical and Treatment-Related Characteristics of PABC Patients

Large, aggressive tumours (28, 31) and advanced stage cancer (25, 26, 31) were more frequent in the PABC group with overexpression of human epidermal growth factor receptor 2 [HER2] (22, 24–28, 30, 31) and oestrogen receptor negative (27, 28) and progesterone receptor negative status (23, 25–28, 30). Seven studies documented information about surgery and treatment. Only five studies reported on surgical procedures in PABC patients. The most common surgical procedure in PABC patients was mastectomy in four studies (23, 27, 28, 30), while one study found breast conservation surgery to be the preferred option (21). Chemotherapy was the chosen regimen of treatment and radiotherapy was observed in two studies (23, 27).

### Survival Outcomes of PABC Patients

Most studies reported on OS, DFS or EFS observed in PABC patients, while only six studies mentioned details related to prognosis (21, 23, 25, 27, 28, 31). Only five studies reported that PABC patients had significantly lower OS (25–27, 31) or DFS (26, 29) than the controls. The 5-year survival rate of women with PABC within 2 years of pregnancy was observed to be comparatively low (64.5% versus 90.6%) compared to that for nulliparous women (31). There was a non-significant difference in OS or DFS or EFS between PABC and controls in six studies (21–24, 28, 30). Only two studies reported that women with PABC had a significantly poor prognosis for survival compared to the controls (27, 31). Women with breast cancer in Japan who had given birth within the previous 2 years had significantly twice the risk of dying compared to nulliparous women (31). Women with breast cancer in South Korea who received adjuvant chemotherapy and were diagnosed within a year after pregnancy or delivery had a significantly poorer prognosis than those in the non-PABC group (27). Four studies reported that pregnancy was a non-significant prognostic factor for survival of breast cancer patients (21, 23, 25, 28) while five studies did not report the information at all (22, 24, 26, 29, 30).

## Discussion

This systematic review found that the poor survival and prognosis of women with PABC compared to their non-pregnant counterparts in Asia were not consistently observed. Less than half of the selected studies supported that woman with PABC has significantly poor survival and prognosis. Generally, studies that reported a non-significant difference of OS had a small sample size and were conducted in a single hospital setting. Our review is supported by a study conducted in the Texas Cancer Centre which reported that women with PABC had statistically non-significant differences in locoregional recurrence, distant metastases or OS compared to those with non-PABC (17). However, that study noticed that pregnancy contributed to a delay in diagnosis and treatment for breast cancer. Women with PABC tend to have advanced cancer, an aggressive feature and an elevated risk of recurrence (13, 16, 17, 21, 25, 28, 29, 32). Patients with PABC were substantially more likely to have hormone-receptor negative tumours (32) as well as being younger at the time of diagnosis than those with non-PABC (23, 25, 28, 30).

There was uncertainty in the management of breast symptoms during pregnancy or the postpartum period, thus a consequent delay in the diagnosis and treatment of breast cancer (33). Some medical practitioners consider breast signs as harmless and related to lactation. Some may be reluctant to order invasive investigations in pregnant or lactating mothers. Raising awareness among women and physicians may help reduce diagnostic delays. Diagnostic tests for breast cancer in pregnancy include ultrasound, fine needle aspiration and mammography with foetal shielding (13).

A meta-analysis of 30 world-wide studies reported that women with PABC significantly had 1.60 times poorer DFS and 1.44 times higher risk of death compared to those with non-PABC (34). Much of the published data from Western countries reported poor prognosis and low survival rates in PABC compared to non-PABC cases (15, 18, 32, 35). A Brazilian study reported a median survival time of 30.1 months for women with PABC, which was significantly shorter than that of the control group (53.1 months) (18). A large population-based Swedish study documented that woman with PABC showed a significantly higher mortality rate than women with non-PABC, after adjusting for age, calendar year, education and region (32). Women with PABC from two medical centres in Colorado, USA had a significantly three times greater risk of distant recurrence or death compared to nulliparous cases (15).

## Conclusion

This review concluded that women with PABC in Asia do not consistently have poor survival outcomes. In view of the increasing prevalence of risk factors for breast cancer and older childbearing age, it is likely that the frequency of PABC will increase. We hope that our systematic review will encourage more clinicians and researchers to investigate PABC in different regions across Asia. The end goal would be to target populations particularly from low- and middle-income countries, as data from these regions are highly limited. Awareness of PABC, and the limitations in diagnosing and treating it, is imperative for all providers who care for women with this diagnosis. Our review also highlights the need to conduct large-scale multinational epidemiological studies focusing on PABC cases across Asia to establish the incidence and survival outcomes in PABC patients. There is a need for more in-depth population-based studies with long-term follow-up to be conducted focusing on PABC cases, comparing their characteristics and outcomes to those of non-PABC cases.

### Strengths and Limitations

To our best knowledge, this is the early review to systematically examine published research on the survival outcomes of PABC patients in Asia. The findings reported are from different countries across Asia; however, it is not possible or appropriate to generalise the findings across the diverse Asian communities because most of these studies were not conducted on large cohorts. Additionally, it only includes articles published in English, thus some relevant studies in other languages have been missed. Furthermore, it was difficult to pool the findings of studies due to differences of PABC definition, comparison groups and survival outcomes. A consensus is needed for the PABC definition. Most studies used retrospective records, thus pregnancies or births might not be documented and verified.

## Figures and Tables

**Table 1:** Review of characteristics and survival-related information of PABC of the selected articles

No	Author (year)	Study design; recruitment period; country	Definition PABC, control and sample size	Median age	Quality assessment	Median (range) follow-up time	SR/OS/DFS/EFS	Death/recurrence/metastasis
1.	Bajpai et al. (2021)	Retrospective cohort; 2013–2020; India, single centre	BC during pregnancy (antepartum) (*n* = 34) BC within 1 year after delivery (postpartum) (*n* = 70)	31 (22–42) years old	Moderate	Antepartum: 38 (1–67) months Postpartum: 24 (0.5–62) months	Median OS: Antepartum: 52.4 months (95% CI: 39.6, NA) Postpartum: not achieved Median EFS: Antepartum: 52.4 months (95% CI: 24.0, NA) Postpartum: not achieved 3-year SR: Antepartum: 74.2% (95% CI: 58.3, 94.4) Postpartum: 62.8% (95% CI: 47.9, 82.3) 3-year EFS: Antepartum: 58.1 (95% CI: 41.6, 81.2) Postpartum: 50.5% (95% CI: 37.3, 68.4)	PABC not associated with worse OS or EFS (PABC was not in the model)
2.	Jin et al. (2021)	Retrospective cohort; 2016–2018; China, 27 hospitals	BC during pregnancy or during lactation Antepartum (*n* = 83) Postpartum (*n* = 81)	33 (24–47) years old	Moderate	36 (3–59) months	OS–P = 0.346 DFS–P = 0.525	LR or Distant metastasis: 11.0% (18/164) patients Death: 3.0% (5/164)
3.	Zhang et al. (2021)	Retrospective cohort; 2012–2017; China, two centres	BC during pregnancy or within 1 year after delivery Antepartum (*n* = 22) Postpartum (*n* = 19) Matched control for stage, age and year diagnosis (*n* = 41)	PABC: 31.6 (24–42) years old Non-PABC: 32.2 (26–42) years old	High	No data	Median OS (*P* = 0.131): -PABC: 82.8 months (95% CI: 39.3, 126.5) -Non-PABC: 80.1 months (95% CI: 56.7, 103.6) Median DFS (*P* = 0.167): -PABC: 29.0 months (95% CI: 6.5, 51.5) -Non-PABC: 40.9 months (95% CI: 22.8, 58.8)	PABC not associated with worse OS or DFS (PABC was not in the model)
4.	Han et al. (2020)	Retrospective cohort; 2005–2018; China hospital-based, single centre	PABC1: Symptoms of BC during pregnancy or lactation (1 year after delivery) of first child (*n* = 79). PABC2: Symptoms of BC during pregnancy or lactation (1 year after delivery) of second, third and subsequent child (*n* = 124).	PABC: 33 (23–46) years old	Moderate	59 (2–144) months	3-year DFS (*P*=0.325): -PABC1 -78.4% -PABC2 - 83.7%	No data
5.	Choi et al. (2019)	Retrospective cohort; 2007–2015; South Korea hospital-based, single centre	Delivery/abortion within 9 months of BC diagnosis (antepartum) (*n* = 18) BC within 1 year of delivery (postpartum) (*n* = 45) Non-PABC (*n* = 3,624)	% age < 35 years old: PABC: 38.1% Non-PABC: 8.7%	High	No data	Unadjusted 5-year SR: -PABC: 80.11% (95% CI: 65.37, 89.07) versus non PABC: 95.99 % (95% CI: 95.26, 96.61; *P* < 0.0001) -Antepartum PABC: 88.89% (95% CI: 43.30, 98.36) versus non-PABC: 95.76% (95% CI: 95.02, 96.40; *P* = 0.6353) -Postpartum BC: 77.15% (95% CI, 60.12, 87.61) vs non PABC: 95.97% (95% CI, 95.24, 96.60; *P* < 0.0001).	Death: -Antepartum: aHR = 1.09 (95% CI: 0.15, 7.91); *P* = 0.1708 -Postpartum: aHR = 1.57 (95% CI: 0.82, 2.99); *P* = 0.1708 Pregnancy after BC - aHR = 0.86 (95% CI: 0.26, 2.83), *P* = 0.8004
6.	Suleman et al. (2019)	Retrospective cohort; 2001–2010; Saudi Arabia hospital-based, single centre	BC during pregnancy (*n* = 110) Time matched non-PABC (*n* = 114)	PABC: 34 (20–45) years old Non-PABC: 44 (24–48) years old	Moderate	PABC: 34 months Non-PABC: 54 months	5-year SR: -Pregnant - 65% -Non-pregnant - 82% (*P* = 0.002) DFS: -Pregnant - 47.5% -Non-pregnant - 65.4% (*P* = 0.002)	No data
7.	Bae et al. (2018)	Retrospective cohort; 1996–2015; South Korea Breast Cancer Society Registry database	PABC - diagnosed within 1 year after pregnancy or delivery (*n* = 411) Non-PABC - BC who were not pregnant and had not delivered within the previous year (*n* = 83,381)	% age < 40 years old PABC: 89.2% Control: 29.4%	High	No data	10-year SR: -PABC who received adjuvant chemotherapy, 70.1% versus non-PABC 86.8% (*P* < 0.001). -PABC who received neoadjuvant chemotherapy, 78.5% versus non-PABC 75.1% (*P* = 0.123)	Death: PABC received adjuvant chemotherapy had poor prognosis than control (aHR 1.63; 95% CI: 1.27, 2.08; *P* < 0.001)
8.	Chuang et al. (2018)	Retrospective cohort; 2002–2014; Taiwan Cancer Registry	Antepartum (*n* = 90); One year postpartum (*n* = 347) > 1– ≤ 2 year postpartum (*n* = 410) > 2– ≤ 5 year postpartum (*n* = 1,583) No pregnancy (*n* = 27,800)	% age < 35 years old Antepartum: 64.4% One year postpartum: 59.1% > 1– ≤ 2 yr postpartum: 42.2% > 2– ≤ 5 yr postpartum: 29.7% No pregnancy: 6.9%	High	Antepartum: 4.28 (1.13, 10.9) years One year postpartum: 4.62 (1.02, 11.1) years >1– ≤ 2 years postpartum: 4.46 years (1.07, 10.8) > 2– ≤ 5 years postpartum: 4.79 (1.21, 10.5) years No pregnancy: 4.91 (1.2, 10.8) years	5-year SR adjusted for age and calendar year: Antepartum: 79.7% One year postpartum: 77.8% > 1– ≤ 2 years postpartum: 83.5% > 2– ≤ 5 years postpartum: 87.0% No pregnancy: 89.5%	Death: Antepartum versus no pregnancy - aHR 1.42 (95% CI: 0.83, 2.45) One year postpartum versus no pregnancy - aHR 1.29 (95% CI: 0.96, 1.74) > 1– ≤ 2 year postpartum versus no pregnancy - aHR 1.27 (95% CI: 0.95, 1.70) > 2– ≤ 5 year postpartum versus no pregnancy - aHR 1.06 (95% CI: 0.88, 1.27)
9.	Strasser-Weippl et al. (2015)	Retrospective cohort; 1990–2012; China, single centre	BC diagnosed within 5 years after a full-term pregnancy (*n* = 109) Non-PABC (*n* = 1,274)	% age ≤ 30 years old PABC: 56.7% Non-PABC: 2%	Moderate	No data	DFS: Recent pregnancy vs no recent pregnancy: aHR 1.624 (95% CI: 1.04, 2.54); *P* = 0.034	No data
10.	Yang et al. (2014)	Retrospective cohort; 1984–2009; Taiwan, single centre hospital-based	Antepartum (*n* = 15) BC within 1 year after delivery - postpartum (*n* = 11) Age-matched non-PABC (*n* = 104)	Antepartum: 33 (25–40) years old Postpartum: 37 (30–41) years old Non-PABC: 40 (25–44) years old	Moderate	No data	5-year SR (*P* = 0.15): Antepartum PABC - 65.7% Postpartum PABC - 81.8% Non-PABC - 90.5% 10-year SR (*P* = 0.45): Antepartum PABC - 56.4% Postpartum PABC - 70.1%	No data
11.	Nagatsuma et al. (2013)	Retrospective cohort; 2000–2007; Japan National Cancer Centre Hospital	Group A: BC women who gave birth within the previous 2 years (*n* = 37) Group B: BC women who gave birth between 3 years and 5 years previously (*n* = 59) Group C: BC women who gave birth more than 5 years previously (*n* = 181) Nulliparous BC women (*n* = 249)	Group A: 35 (26–44) years old Group B: 37 (27–43) years old Group C: 41 (32–44) years old Nulliparous: 38 (22–44) years old	High	6.3 years (range: 0.1, 11.7 years)	5-year SR (*P* < 0.001): Group A - 64.5% Group B - 79.3% Group C - 88.2% Nulliparous - 90.6%	Death: Group A versus nulliparous group aHR = 2.19 (95% CI: 1.05, 4.56); (*P* = 0.0364)

Notes: BC = breast cancer; PABC = pregnancy-associated breast cancer; IQR = interquartile range; CI = confidence interval; SR = survival rate; DFS = disease-free survival; EFS = event-free survival; LR = local recurrence; OS = overall survival; aHR = adjusted hazard ratio
